# Pluripotent muse cells derived from human adipose tissue: a new perspective on regenerative medicine and cell therapy

**DOI:** 10.1186/2001-1326-3-12

**Published:** 2014-05-22

**Authors:** Ariel A Simerman, Daniel A Dumesic, Gregorio D Chazenbalk

**Affiliations:** 1 Department of Obstetrics and Gynecology, David Geffen School of Medicine at the University of California, 10833 Le Conte Ave, Box 951740, Los Angeles, CA 90095-1740, USA

**Keywords:** Adult pluripotent stem cells, Muse cells, Non-tumorigenic, Quiescence, Regenerative medicine

## Abstract

In 2010, Multilineage Differentiating Stress Enduring (Muse) cells were introduced to the scientific community, offering potential resolution to the issue of teratoma formation that plagues both embryonic stem (ES) and induced pluripotent (iPS) stem cells. Isolated from human bone marrow, dermal fibroblasts, adipose tissue and commercially available adipose stem cells (ASCs) under severe cellular stress conditions, Muse cells self-renew in a controlled manner and do not form teratomas when injected into immune-deficient mice. Furthermore, Muse cells express classic pluripotency markers and differentiate into cells from the three embryonic germ layers both spontaneously and under media-specific induction. When transplanted *in vivo*, Muse cells contribute to tissue generation and repair. This review delves into the aspects of Muse cells that set them apart from ES, iPS, and various reported adult pluripotent stem cell lines, with specific emphasis on Muse cells derived from adipose tissue (Muse-AT), and their potential to revolutionize the field of regenerative medicine and stem cell therapy.

## Review

### Embryonic and induced pluripotent stem cells: the gold standard

ES cells have unequivocally taken center stage in the field of stem cell research. Isolated from human blastocysts in the late 20th century, ES cells exhibited the potential to treat a plethora of previously irreversible disorders through their capacity to generate tissues of the three embryonic germlines, and thus to revolutionize regenerative medicine [1-3]. Their unlimited proliferation as well as their ability to remain in an undifferentiated state for extended periods of time secured their position at the forefront of scientific research for over two decades. However, evidence has since emerged that ES cells exhibit high rates of immunorejection upon transplantation and form teratomas as a result of their unbridled proliferation [4]. In conjunction with debates surrounding the bioethical issues concerning the usage of human embryos, this teratogenic propensity precludes the practical application of ES cells in regenerative medicine.

In response, iPS cells quickly assumed their position as the new, fervently pursued subject of interest in the stem cell field [5,6]. iPS cells are reprogrammable through the induction of “Yamanaka factors,” including Nanog, Oct3/4, Sox2, c-Myc and Klf4, and can be utilized autologously, resolving issues of immunorejection at the time of transplantation [6-9]. While iPS cells resolve the bioethical concerns surrounding the use of stem cells extracted from human embryos, the production of teratomas as a consequence of uncontrolled cell proliferation impede the translational application of these cells for stem cell therapy [10,11]. Furthermore, mature iPS cells possess an epigenetic memory, wherein the remnants of posttranslational histone and DNA modifications prevent complete reprogramming as well as their physiological function beyond the range of their cell type of origin [7,12-14]. Investigators have made attempts to address these issues, but to little avail [15,16]. Despite excessive monetary and temporal efforts devoted to the study of both ES cells and iPS cells, there has been little progress made in overcoming the hurdles facing these stem cells and their use towards cell therapy.

### Adult pluripotent stem cells at a glance

Other, non-reprogrammed pluripotent stem cell populations have caught the attention of the scientific community as an alternative to ethically contentious ES cells and genetically modified iPS cells. However, though several populations of adult stem cells that possess pluripotency have been put forth, many have faced a great deal of suspicion due to irreproducibility and pluripotency marker identification. Isolated from bone marrow, multipotent adult progenitor cells (MAPCs), both pluripotent and non-tumorigenic, were reported to contribute to chimeric offspring when injected into a mouse model and to regenerate damaged tissue *in vivo*[17,18]. Human marrow-isolated adult multilineage inducible (MIAMI) cells and very small embryonic-like stem cells (VSELs), isolated from umbilical cord blood in addition to bone marrow, were soon to follow, exhibiting similar pluripotent and non-tumorigenic properties [17-19]. Like VSELs, unrestricted somatic stem cells (USSCs), isolated from umbilical cord blood, are reportedly pluripotent but lack the classic pluripotent stem cell marker expression [20]. These adult pluripotent stem cell lines have all been publically flagged for further investigation and reproduction, or in the case of VSELs, negated entirely [21,22].

Stimulus-triggered acquisition of pluripotency (STAP), characterized by exposing splenic CD45+ lymphocytes to acidic conditions followed by incubation with leukaemia inhibitory factor (LIF), has recently been described as a method of bestowing pluripotency upon somatic cells [23]. However, STAP cells form teratomas, hindering their clinical application. STAP cells are currently under investigation to determine the overall validity of the published results as well as the mechanism behind their reprogramming.

### The advent of muse cells

In 2010, a research team at the Tohoku University in Sendai, Japan, identified a population of pluripotent mesenchymal stem cells (MSCs), named Multilineage Differentiating Stress Enduring (Muse) cells, through the induction of severe cellular stress. Initially isolated from bone marrow aspirates and human skin fibroblasts, this cell population expresses the pluripotency marker stage-specific embryonic antigen-3 (SSEA-3) as well as the mesenchymal cell marker CD105 [24,25]. Our research team at the University of California, Los Angeles, discovered that Muse cells exist in human adipose tissue as well [26]. Imposing alternate, yet comparably high stress conditions, we isolated Muse cells, derived from adipose tissue harvested by lipoaspiration (Muse-AT cells) [26]. More recently, investigators have shown that Muse cells can be isolated from commercially available human adipose stem cells (ASCs) through SSEA-3 cell sorting as well [27]. Much like their relatives derived from bone marrow aspirates and skin fibroblasts [24], Muse-AT cells form cell clusters similar to ES cells when grown in suspension. Muse-AT cells also express the classic pluripotency markers SSEA3, Sox2, Oct3/4, Nanog and TRA1-81 [24,26].

### Pluripotency of muse cells *in vitro* and *in vivo*

Muse cells grow in suspension as cell clusters reminiscent of embryonic stem cells. Muse cells intrinsically express classic pluripotency markers including SSEA3, Nanog, Oct3/4, Sox2, TRA1-60 and TRA1-81 [24,26]. Furthermore, Muse cells, both spontaneously and under specific culture conditions, express mesodermal (α-smooth muscle actin, desmin, DLK, Bodipy and myosin D), endodermal (α-fetoprotein, cytokeratin 7, GATA6 and pan keratin), and ectodermal (neurofilament-M, MAP2, Glut-R, and NeuroD) markers [24,26]. Intravenously administered GFP-labeled human Muse cells injected into damaged skin, muscle, and liver tissue of immunodeficient mice were able to integrate and differentiate accordingly *in vivo* and contribute vastly to tissue regeneration within 2–4 weeks [24]. Human Muse cells were successfully applied in models of fulminant hepatitis, muscle degeneration and skin injury in different mouse-disease models [21].

Muse cells, induced to grow into 3D cultured skin *in vitro*, have the capacity to give rise to mature melanocytes, contributing to tissue regeneration after engraftment into damaged skin [28]. The authenticity of Muse-derived melanocytes is supported both molecularly and morphologically. Muse-derived melanocytes were pigmented, as compared to the negative control. Furthermore, they differentially expressed human melanocyte markers. This evidence suggests that various skin diseases due to melanocyte dysfunction, including the prevalent vitiligo, could be treated using Muse-derived melanocyte transplantations.

### Muse cells and non-tumorigenicity: a scientific anomaly

The definition of pluripotency relies upon a cell’s ability to differentiate into the three embryonic germ layers, and in the case of stem cells, to self-renew [29]. Tumor formation coincides with both pluripotency and self-renewal and has emerged as a critical factor in determining the pluripotent capacities of both ES and iPS cells [30-33]. However, as seen in ES and iPS cells, the capacity for tripoblastic differentiation and self-renewal is frequently uncontrolled, and often materializes in teratoma formation, hindering the exploitation of their pluripotency for regenerative purposes. However, innate pluripotent stem cells, including epiblast stem cells and neoblasts present in planarians, do not form teratomas in the way that ES and iPS cells do [34]. Planarians and newts, which have the ability to regenerate amputated body parts, have also been shown to possess pluripotent cells [35].

**Figure 1:**
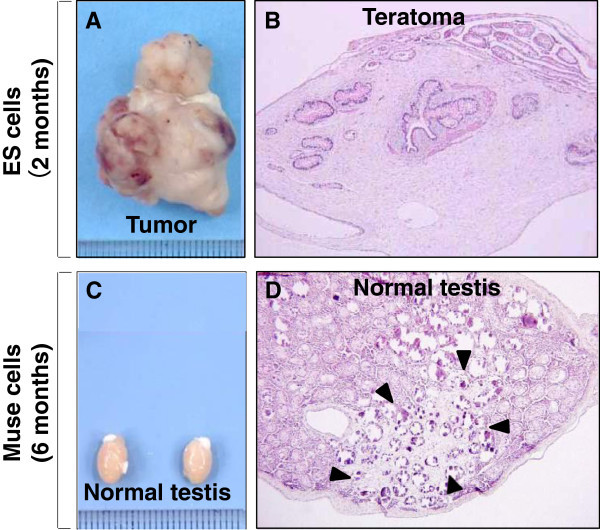
**Non-tumorigenicity of Muse cells. (A)** Embryonic stem (ES) cells infused into immunodeficient mice (SCID mice) testes, formed teratomas within 8 to 12 weeks, **(B)** Histological analysis showed that the teratoma contained muscle tissue, intestine-like structures and keratinized skin, **(C)** Muse cell-transplanted testes did not generate teratomas similar to untreated testes, and **(D)** Testes injected with Muse cells maintained normal structure even 6 months post-injection (Pictures reproduced from *Proc Natl Acad Sci USA* 2010, 107: 8639–43, and *Stem Cells Dev* 2014 DOI: 10.1089/scd.2013.0473).

Despite their characterization as pluripotent stem cells, Muse cells exhibit both low proliferative and telomerase activities, a normal karyotype as well as asymmetric division, and thus do not undergo tumorigenesis or teratoma formation when transplanted into a host organism [24,26,27] (Figure 1). Low telomerase activity is partially responsible for this peculiar balance [27]. Muse cells also exhibit much lower expression of the so-called “Yamanaka factors” in comparison with iPS cells (>10^5^ fold decrease) [25]. Furthermore, Muse cells exhibit a slight increase in expression of Sox2, Nanog, Oct3/4 as compared to non-muse cells (2–4 fold) [24,26]. Intermediate expression of the genes that have been shown to confer pluripotency and teratogenesis may explain how Muse cells retain their lineage plasticity while simultaneously negating teratoma formation.

Lin28, a RNA-binding protein gene, maintains both pluripotency and tumorigenesis in ES and iPS cells [36,37]. Let-7, a microRNA that regulates embryonic development, cell differentiation and tumor suppression, has the opposite effect [37]. While over-expression of Let-7 blocks Lin28 gene expression, Lin28 expression degrades Let-7, maintaining a balance in their expression, controlling development and disease [37]. Levels of Lin28 expression decline over the course of embryonic development while let-7 miRNA’s simultaneously increase, suppressing self-renewal of undifferentiated cells and stimulating cell differentiation. ES and iPS cells have a very high Lin28/Let7 ratio, which has been thought to play a major role in their tumorigenic propensities [37]. In the absence of a strong Lin28 influence, Muse cells retain their pluripotent capacity [25]. Over-expression of Let-7 in Muse cells would potentially play a critical role in inhibiting Lin28 expression, and therefore would protect these cells from tumorigenic proliferation and teratoma formation after *in vivo* transplantation.

Retaining their self-renewing ability, Muse cells do not undergo unbridled proliferation or tumor formation *in vivo*, setting them apart from ES cells. When transplanted into the testes of immune-deficient mice, Muse cells did not form teratomas while ES cells formed large teratomas within 8–10 weeks [27]. As pluripotency and tumorigenesis have commonly been considered a packaged deal, it is imperative to consider what factors allow Muse cells to avoid tumor formation while retaining their capacity for differentiation into all three germ lineages.

It has been postulated that iPS cells are generated exclusively from Muse cells [25]. When fibroblasts were subjected to the Yamanaka factors, only Muse cells underwent successful iPS cell generation [25]. Gene analysis shows that classical markers of tumorigenesis, including BCR1, CCMB1 and CCMB2, are highly expressed in iPS cells derived from Muse cells as compared to naïve Muse cells [25]. In contrast, CDKN1A and CDKN2A, involved in tumor suppression, are highly expressed in Muse cells versus iPS cells derived from Muse cells [25]. This may shed light on the effects of the induction of the Yamanaka factors and their contribution to tumorigenesis, as well as the inherent propensity for teratoma formation in iPS cells but not Muse cells, however further studies are required to elucidate this distinction.

### Muse cells derived from adipose tissue (Muse-AT)

**Figure 2:**
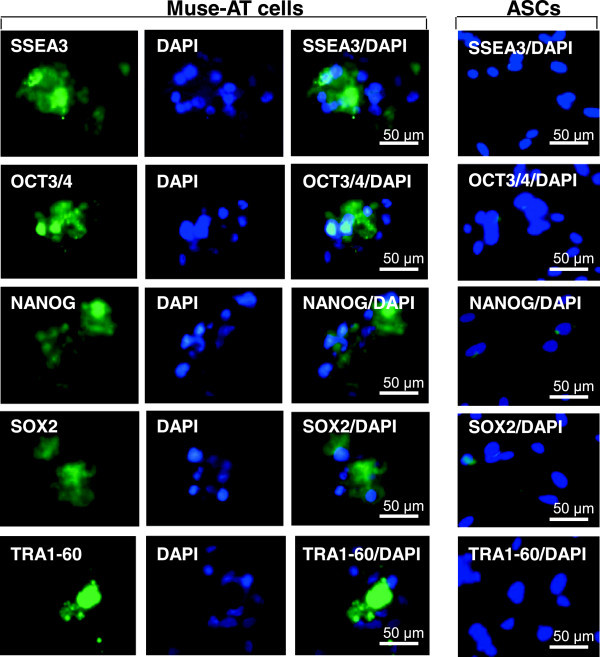
**Muse-ATs express pluripotent stem cell markers.** Immunofluorescence microscopy demonstrates that Muse-AT aggregates, along with individual Muse-AT cells, express characteristic pluripotent stem cell markers, including SSEA3, Oct3/4, Nanog, Sox2, and TRA-1-60 [24,26] (Figure 2). Comparatively, ASCs (right panel) derived from the same lipoaspirate under standard conditions [38] were negative for these pluripotent stem cell markers. Nuclei were stained with DAPI (blue). Original magnification, 600 X (Pictures reproduced from *PLoS One* 2013, 8(6):e64752).

We isolated Muse cells derived from adipose tissue harvested by lipoaspiration (Muse-AT cells) under severe cellular stress conditions [26]. Furthermore, Muse cells isolated from adipose tissue can be also obtained from commercially available human adipose stem cells (ASCs) through SSEA-3 cell sorting [27]. Much like their relatives derived from bone marrow aspirates and skin fibroblasts [24], Muse-AT cells grow in suspension as cell clusters, similar to embryoid bodies, which express the classic pluripotency markers SSEA3, Sox2, Oct3/4, Nanog, and TRA1-60 [24,26] (Figure 2).

**Figure 3:**
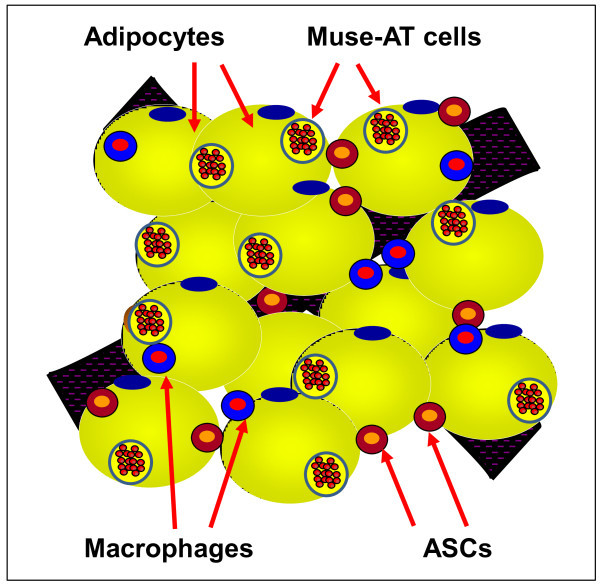
**Graphical depiction of different cell components present in adipose tissue.** Adipose tissue is composed of adipocytes and the stromal vascular fraction containing adipose tissue macrophages (ATMs), adipose stem cells (ASCs) and Muse-AT cells, among other cell components.

Under unperturbed physiological circumstances, Muse-AT cells reside within the adipocyte and stromal vascular fractions [26]. Within both fractions, cross-talk between ASCs and adipose tissue-residing macrophages (ATMs) contributes to cell plasticity, adipogenesis and ASC formation [39] (Figure 3). ASCs, ATMs and adipose immune infiltrating cells may interact with neighboring Muse-AT cells, affecting their lineage plasticity, adipose tissue differentiation and repair, and the production and recruitment of signaling molecules in times of cellular stress [26].

**Figure 4:**
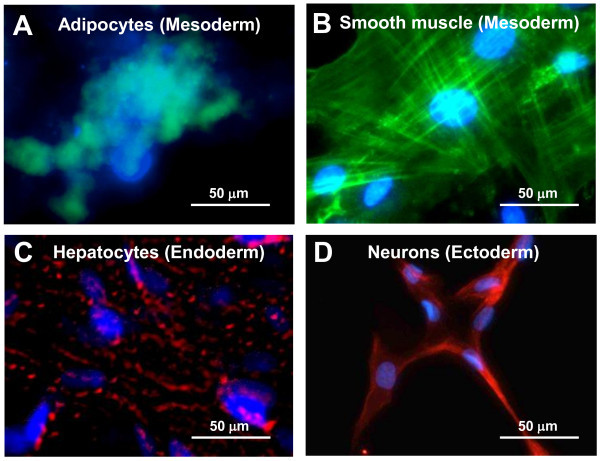
**Tripoblastic characteristics of Muse-AT cells.** Muse-AT cells were grown as adherent cells in the presence of **(A)** adipogenic medium; the formation of adipocytes was detected using BODYI-PI-C16 which identify lipid drops present in adipocytes; **(B)** myogenic differentiation medium; the formation of myocytes was detected using an anti-human MSA antibody; **(C)** hepatogenic differentiation medium; formation of hepatocytes was detected using an anti-cytokeratin 7 antibody; **(D)** Muse-AT cells were grown for 7 days as non-adherent cells and then cultured for an additional 7 days as adherent cells; neural-like cells were detected by immunofluorescence using an anti-human MAP2 antibody. Nuclei were stained with DAPI (blue). Original magnification was 600X. (Pictures reproduced from *PLoS One* 2013, 8(6):e64752).

Muse-AT cells differentiate into mesodermal, endodermal and ectodermal embryonic germ lineages spontaneously, with 23%, 20% and 22% respective efficiencies [26]. Incubated in the presence of lineage-specific media, Muse-AT cells differentiate with 82%, 75% and 78% respective efficiencies. Furthermore, Muse-AT cells exhibit lineage-specific morphological characteristics after only 3 days in culture [26]. Immunocytochemistry studies showed expression of markers for adipocytes, myocytes, hepatocytes and neural cells in both naïve Muse cells and Muse cells that had been induced to differentiation in tissue-specific culture media [26]. For example, Muse-AT cells demonstrated formation of lipid droplets when induced to differentiate into adipocytes (Figure 4A), as well as characteristic smooth muscle striations when induced to differentiate into myocytes (Figure 4B) [26]. Employing identical culture conditions used to induce ES and iPS differentiation into hepatocytes, Muse-AT cells were also driven to differentiate into hepatocyte-like cells (Figure 4C) [26]. Furthermore, Muse-AT cells differentiate into neural-like cells, forming long, finger-like projections, typical of neurons, similar to ES and iPS cells (Figure 4D) [26]. Muse-AT cells could therefore be applied to treat muscle, liver and brain disorders without the teratogenic risk associated with ES and iPS cells.

Genes associated with cell death and survival, embryonic development, organismal development, tissue development, cellular assembly and organization, and cellular function and maintenance are highly conserved, with homologues present in numerous primordial organisms including yeast (*S. Cerevisiae*), *C. elegans*, *chlamydomonas*, *T. californica*, and fruit fly (*Drosophila*). Muse-AT cells differentially express genes from all of the aforementioned categories, suggesting that Muse cells may function according to a highly conserved cellular mechanism related to cell survival in response to severe cellular stress [40,41].

For medical and cosmetic reasons, lipoaspirate material is routinely extracted from the human body due to its availability, accessibility and abundance. Because adipose tissue is present in such a high abundance within the human body, the number of extractable Muse-AT cells is abundant compared to that which can be extracted from bone marrow or dermis. Muse-AT cell isolation from lipoaspirate material is both temporally and monetarily efficient.

### Muse-AT cells and quiescence

Muse cells exist in a quiescent state under normal physiological circumstances within the cellular niche prior to cellular stress disruption [24,26]. Contributing to their capacity for self-renewal, multiple adult stem cell lineages have been shown to exist in a quiescent state, including hematopoietic stem cells and epithelial stem cells [42,43]. The mobilization of quiescent stem cells is attributed to CXCL2, a chemokine, which functions in stem cell homing [43]. Exposing MSCs to CXCL2 prior to transplantation increases post-transplant stem cell survival rates in cases of myocardial infarction [44]. In the case of Muse-AT cells, CXCL2 is expressed 770 folds higher than in neighboring ASCs, which could therefore explain their genetic predisposition to cellular stress resistance [22]. CXCL2, overexpressed in cancer cells, contributes both to cancer cell survival and malignancy. Quiescence is the “natural” state in which Muse cells exist. On the other hand, quiescence contributes to the maintenance of malignancy in cancer stem cells in a dormant stage, susceptible to relapse in the wake of cancer treatment [36]. The internal/external stimuli that activate Muse cells (prolonged treatment with proteolytic enzymes, lack of nutrients, low temperatures, hypoxia) are entirely different than those that activate cancer stem cells (ionizing radiation, ultraviolet radiation, chemical compounds, reactive oxygen species, error prone DNA repair, among others).

Nevertheless, Muse cells (non-tumorigenic cells) can be converted into iPS cells (tumorigenic cells) when exposed to the four “Yamanaka factors” [25].

Microarray analysis showed differential expression of 144 critical genes involved in cell death and survival (e.g. SGK1, MDH1, ATF2, HSPA8, PDIA3, BRD1, CALM1, NR4A2, GATA2, CDK6, NUF2, CDK6, BRC1, BUB1B and CXCL2), suggesting that expression of these genes could contribute to Muse-AT cell activation from quiescence [26]. Furthermore, Muse-AT cells over-express *ALDH1A2* (47 fold change versus ASCs) and *SOD2* (41 fold change versus ASCs) which boast anti-oxidative stress and anti-apoptotic functions [44,45]. Interestingly, DNA repair genes are generally up-regulated in Muse-AT cells, indicating a high capacity to resist DNA damage due to cellular stress [26].

The application of stem cells in regenerative medicine has often been impeded by a low survival rate (<3%), when exposed to the high stress environment of the engraftment site, especially in cases of myocardial infarction, ischemic injury and stroke [40,46-50]. Investigators have utilized hypoxia preconditioning (HPC), a process in which stem cells are introduced to hypoxic conditions for 24–48 hours prior to transplantation, to acclimatize stem cells to pro-apoptotic factors including hypoxia, malnutrition, pro-inflammatory cytokines and reactive oxygen and nitrogen species, in order to enhance post-transplantation stem cell survival [51-53]. Muse cells, however, do not require HPC or any other pre-transplantation conditioning, as they are intrinsically resilient to cellular stress [24,26]. A long-term trypsin (LTT) incubation, or proteolytic digestion, was employed to isolate Muse cells from bone marrow aspirates and skin fibroblasts [24]. Human adipose tissue lipoaspirate material was subjected to even more severe cellular stress conditions including long-term exposure to the proteolytic enzyme collagenase (LTC), serum deprivation, low temperatures and hypoxia. This, in turn, produced a highly purified population of Muse (Muse-AT) cells [26]. As cellular stress induction is imperative to Muse cell isolation, and thus intrinsic to their activation, their capacity for survival and the ultimate translational objective of tissue regeneration *in vivo* is greater as compared to alternative stem cell populations.

## Conclusions

The potential application of Muse cells in regenerative stem cell therapies is both innovative and promising. Muse cells are inherently resistant to cellular stress, and genetically resilient to DNA damage, supporting their application for the investigation of age-related and degenerative diseases. Moreover, because Muse cells possess a pre-perturbation and an intrinsic propensity for quiescence, they may have the potential to elucidate new avenues of cancer research, specifically with regards to the mechanism behind quiescence, and malignancy [26,43]. Finally, and perhaps most thrillingly, Muse cells could be harvested for the purposes of creating autologous stem cell banks. Because of their differentiation capacity, Muse cells could be utilized to regenerate any type of tissue and thus treat neurological and immune disorders, and injuries to critical organs such as the heart and brain.

Furthermore, Muse-AT cells are isolated from lipoaspirate material which is easily accessible, abundant, and non-invasively extracted from the human body for both medical and cosmetic purposes. Hundreds of millions of adipose cells can be extracted from a mere 1–2 liters of tissue, enhancing the number of extractable Muse-AT cells. Muse-AT cells are a promising candidate for translational application in stem cell therapy and regenerative medicine, thus it is imperative to further investigate and exploit their unique qualities and vast potential.

## Competing interests

The authors declare that they have no competing interests.

## Authors’ contribution

AAS, DAD, and GDC contributed to the writing of this review. All authors read and approved the final manuscript.
